# Efficient degradation of tylosin by *Kurthia gibsonii* TYL-A1: performance, pathway, and genomics study

**DOI:** 10.1128/spectrum.00025-25

**Published:** 2025-04-29

**Authors:** Ye Wang, Boyu Zhao, Jingyi Zhang, Lingcong Kong, Inam Muhammad, Xiaojun Liang, Xiuzhen Yu, Yunhang Gao

**Affiliations:** 1 College of Veterinary Medicine, Jilin Agricultural University 85112, Changchun, China; 2 Department of Zoology, Shaheed Benazir Bhutto University Sheringal, Dir Upper, Pakistan; 3 Institute of Animal Science, Ningxia Academy of Agriculture and Forestry Sciences 205388, Yinchuan, Ningxia, China; 4 Agricultural Mechanization Research Institute, Xinjiang Academy of Agricultural Sciences 74608, Ürümqi, China; USDA-ARS San Joaquin Valley Agricultural Sciences Center, Parlier, California, USA

**Keywords:** TYL, biodegradation, degradation products, degradation pathway, genome analysis

## Abstract

Tylosin is a commonly used macrolide antibiotic, which is commonly utilized in livestock; its release through animal excrement can have detrimental environmental effects. Biodegradation of tylosin (TYL) is an effective bioremediation method. In this study, we identified a novel and efficient TYL-degrading bacterial strain, *Kurthia gibsonii* TYL-A1, capable of degrading 75 mg/L of TYL within 5 days at 30°C, pH 7, with 3% inoculum and yeast extract as the nitrogen source. The bacterium degraded 99% of 75 mg/L TYL in 5 days. Both intracellular and extracellular enzymes collaborated to degrade TYL. Metabolites were analyzed by liquid chromatography-mass spectrometry (LC-MS), revealing that strain TYL-A1 could remove mycophenolic sugar, cleave the ester bond, and further degrade TYL into smaller molecules. The toxicity of the degradation products was lower than that of the parent compound and its natural degradation products. Whole-genome sequencing results indicated that genes encoding glycoside hydrolases and glycosyltransferases, along with metabolism-related genes, were involved in TYL degradation. This study elucidated the degradation mechanism of TYL and highlighted the potential of strain TYL-A1 to remove TYL from the environment.

**IMPORTANCE:**

Tylosin (TYL) contamination has become a hot issue, and microbial removal systems have been widely considered as an economical and environmentally friendly alternative. Our study proposed a new TYL degradation pathway through the biological metabolic pathway of LC-MS metabolite analysis. Whole-genome sequencing further provided the genetic mechanism involved in the degradation process and explained the degradation effect of strain TYL-A1 on TYL. The application of TYL-A1 to actual wastewater highlights the practical relevance of TYL pollution in the environment. This application highlights the importance of microbial germplasm resources in the bioremediation of TYL-contaminated ecosystems. All in all, our study provides a theoretical basis for reducing the pollution of antibiotics in the environment and promoting the sustainable development of the ecological environment.

## INTRODUCTION

Antibiotics are commonly utilized as antibacterial medications and growth enhancers in aquaculture and medicine (1). In 2017, the top 10 nations in terms of veterinary antibiotic consumption were China, Brazil, the United States, Thailand, India, Iran, Spain, Russia, Mexico, and Argentina (2). China, accounting for 45% of the world’s antibiotic usage, is the largest user. The global veterinary antibiotic use was 9,309 tons in 2017, and by 2030, this number is predicted to increase by 11.5% to 10,4079 tons (3, 4). Although antibiotics are now banned as feed additives, a significant number are still used for the prevention and treatment of livestock and poultry diseases. These include macrolides, fluoroquinolones, tetracyclines, and sulfonamides (5). Among them, tylosin (TYL), a broad-spectrum veterinary antibiotic belonging to the macrolides class and produced by *Streptomyces fusiformis*, is the drug of choice for mycoplasma diseases in livestock and poultry (6). Due to the misuse of antibiotics and their structural stability, many non-inactivated antibiotics are excreted through fecal waste, causing environmental pollution (4). Studies have calculated that in Mexican pig farms, approximately 284.3 µg of TYL per kilogram of pig is released into the environment through wastewater (7). TYL is also detected in broilers (8) and in the feces of dairy cows and pigs (9). Antibiotics can enter the body through various routes, with food being the main source (10). The accumulation of antibiotics in the body can cause a series of pathological changes, such as organ allergies and immune suppression. The extensive use of antibiotics greatly impacts biological systems and microbial communities, accelerating the formation of drug-resistant pathogens (antibiotic-resistant bacteria) and resistance genes (antibiotic resistance genes, ARGs) (11, 12).

Antibiotics can currently be eliminated from the environment using various techniques, including the adsorption method (13, 14), advanced oxidation method (15), such as photocatalytic oxidation (16, 17), ozone oxidation (18, 19), persulfate oxidation (20, 21), electrochemical methods (22, 23), and Fenton oxidation (24, 25). Biological repair methods such as phytoremediation (26, 27) and microbial degradation (28) are also employed. However, the physical adsorption method can be limited by the characteristics of the adsorbent and may not completely purify the pollution, requiring a combination with other methods (29). Moreover, some adsorption materials may detect emerging contaminants or release antibiotics during the adsorption process (30). The advanced oxidation method is costly and needs improved adaptability and practicality for real-world applications. Phytoremediation is effective for bioremediating wastewater with antibiotics, but selecting plants with multiple pollution tolerances for actual environments and studying their migration pathways and degradation mechanisms is necessary. Microbial degradation is a safe and effective environmental remediation method where drug-resistant bacteria play a crucial role. Drug-resistant bacteria with degradation functions are isolated through screening, enrichment, and domestication. The enzymes produced by these bacteria can break the chemical bonds in antibiotics, altering their physical structure and thereby reducing or inactivating their activity (31). For instance, the bacterial strain H38 can degrade 5 mg/L of sulfamethazine within 3 days, as isolated from soil by Dong et al. (32). Wang et al. (33) isolated a fungal strain, M503, which effectively degrades tetracycline, doxycycline, and chlortetracycline (CTC). Another study by Tan et al. (34) demonstrated that the bacterial strain TC931, isolated from feces, removed 87.38% of tetracycline. Compared with other methods, microbial antibiotic degradation is relatively simple, easy to control and operate, highly specific, and rarely causes secondary pollution. It is the most economical and effective degradation method currently available and remains a research hotspot.

Currently, there are few microorganisms known to have TYL degradation capabilities, and the mechanisms underlying this process are seldom reported. In this study, a strain with previously unreported TYL degradation functionality was selected, and its degradation performance as well as biodegradation products were evaluated. Additionally, the whole genome of the bacterial strain was sequenced, and the sequencing data were analyzed to identify potential key genes involved in the biodegradation process. This approach aims to enrich the biological resources for TYL degradation and provide a reference for understanding the degradation mechanisms of TYL. Furthermore, the study investigated the strain’s degradation performance in the presence of different metal ions and applied strain simulation to aquaculture wastewater to establish a foundation for future practical applications.

## MATERIALS AND METHODS

### Chemicals and media

TYL was purchased from Shanghai Yuanye Biotechnology Co. Methanol (99.9% purity) and acetonitrile (99.95% purity) were obtained from Fisher Scientific Co. All other chemicals used in the experiment were of analytical grade. The mineral salt medium (MSM, g/L) was prepared with the following composition: MgSO_4_ · H_2_O (0.20 g), KH_2_PO_4_ (0.5 g), K_2_HPO_4_ (1.5 g), NaCl (1 g), and yeast extract (1.0 g). The Luria-Bertani (LB) medium was composed of peptone (10.0 g), NaCl (10.0 g), and yeast extract (5.0 g). The LB medium was prepared by dissolving these ingredients in distilled water. In the degradation experiments, the TYL concentration was 75 mg/L unless otherwise specified. TYL residues were determined using high-performance liquid chromatography with a Shimadzu LC-2030 Plus (Kyoto, Japan). Chromatographic conditions were referenced from Zhao et al. (35).


(1) $$Degradation\;rate\;\left(\%\right)=\left(C_{0}-C_{t}\right)/C_{0}\times 100\%,$$

where *C*_0_ represents the initial concentration of TYL (mg/L), and *C*_*t*_ denotes the concentration of TYL remaining in the solution at time (*t*).

### Degradative properties of TYL-A1

According to our previous study, the strain TYL-A1 was isolated from the soil and identified as *Kurthia gibsonii* TYL-A1 (35). To prepare the strain for degradation experiments, it was inoculated into fresh tryptic soy broth (TSB) for resuscitation. After pre-culturing, the TYL-A1 culture was centrifuged at 4°C and 8,500 rpm for 15 minutes, washed three times with sterile phosphate buffered saline (PBS), resuspended, and adjusted to an optical density (OD_600_) of 2.0 for subsequent degradation experiments. A one-factor experimental design was used to optimize TYL degradation. Degradation experiments were conducted under varying conditions including different nitrogen sources (1 g/L ammonium chloride, ammonium sulfate, ammonium nitrate, urea, and yeast powder), different initial TYL concentrations (25, 50, 75, 100, 125 mg/L), different culture temperatures (20°C, 25°C, 30°C, 35°C, 40°C), different initial pH (3, 5, 7, 9, 11), different bacterial inoculum (1%, 3%, 5%, 7%, 9% vol/vol), and different metal ions (10 mg/L Fe^2+^, Fe^3+^, Cu^2+^, Ca^2+^, Mn^2 +^, Zn^2 +^). TYL concentration was measured using high-performance liquid chromatography (HPLC).

### Degradation kinetics of TYL-A1

Based on the results of the single-factor optimization trial, the optimal culture conditions were established. The degradation kinetics were analyzed by fitting kinetic curves to the observed data. The degradation kinetic curves were plotted as ln (*C*_*t*_/*C*_0_) versus *t* (h). Biodegradation processes typically follow first-order reaction kinetics, described by the following equation:


(2) $$C_{t}=C_{0}\times exp\left(-kt\right).$$

The following formula was used to determine the half-life of the analyte:


 (3) $$t_{1/2}=-ln2/k,$$

where *C*_0_ (mg/L) and *C*_*t*_ (mg/L) represent the analyte concentrations at times zero and *t* (h), respectively, and *k* (h^-1^) is the rate constant for TYL degradation.

### Substrate utilization pattern of TYL-A1

The degradation of TYL by the bacterium TYL-A1 is influenced by the biological activity of the bacteria and their ability to utilize TYL as a carbon source. To assess the substrate utilization patterns, a Biolog ecological plate (Biolog, Inc., Hayward, CA, USA) was employed, with each well containing a different single carbon source. The plate was divided into six categories and includes 31 carbon substrates: 12 carbohydrates, 6 amino acids, 4 polymers, 5 carboxylic acids, 2 phenolic acids, and 2 amines and amides. This setup is used to characterize the carbon source utilization patterns of pure or mixed bacteria cultures and allows comparison of the utilization effects of six types of carbon sources. The experimental method was adapted from Feng et al. (36), with TYL added to achieve a final concentration of TYL of either 0 or 75 mg/L. Incubation was performed at 30°C, protected from light, and the development of color was monitored continuously at 590 nm using a microplate reader for 10 days. The average well color development (AWCD) was calculated, and the degree of carbon source utilization by TYL-A1 was determined based on the OD_590_ and the AWCD measurements.


AWCD =∑ODi/31,

where ODi is the optical density per hole.

### Degradative enzyme localization assay

To determine whether the degradation of TYL is mediated by extracellular or intracellular enzymes, a TYL-A1 bacterial suspension was cultured in 200 mL of inorganic salt medium at 30°C for 2 days. The culture was then centrifuged at 8,500 rpm for 15 minutes at 4°C to separate the supernatant, which was expected to be rich in extracellular enzymes. Following this, the bacterial pellet was resuspended in PBS and centrifuged again at 8,500 rpm for 15 minutes at 4°C. This process was repeated twice to obtain a purified bacterial pellet. The pellet was then resuspended in 200 mL of PBS, and the cell walls were disrupted using an ultrasonic cell disruptor to release intracellular enzymes. The ultrasonic treatment parameters were set as follows: a total cycle time of 20 minutes, with an on time of 4 seconds and an off time of 3 seconds. Both intracellular and extracellular enzyme preparations were added to PBS containing 75 mg/L of TYL under optimal conditions. The residual concentration of TYL was subsequently measured using HPLC.

### Characterization of degradation products

A TYL-A1 bacterial suspension was inoculated at 3% (vol/vol) in 100 mL of a medium containing 75 mg/L TYL. The culture was incubated at 30°C, pH 7, with shaking at 135 rpm and protected from light. Samples of 200 µL were collected at 12 hours and 4 days. To each sample, 800 µL of extraction solvent (methanol:acetonitrile = 1:1 [vol/vol]) was added, and the mixture was vortexed for 30 seconds followed by low-temperature ultrasonic extraction at 5°C for 30 minutes. To re-dissolve the residue, 120 µL of a solvent mixture (acetonitrile:water = 1:1) was added to re-dissolve the solution. This extraction process was repeated twice, and finally, 20 µL of the supernatant was analyzed. Chromatographic conditions were referenced from Li et al. (37). Mass spectrometry was performed in positive ion mode after ionization using electrospray ionization. The mobile phases used are listed in Table S1.

### Toxicity analysis of degradation products

*Escherichia coli* and *Staphylococcus aureus* were resuscitated and grown in LB broth, and bacterial suspensions were prepared by adjusting the OD_600_ = 0.5. The experiment consisted of three groups: the positive control group (PC), the negative control group (NC), and the biodegradation product group (TC). Strain TYL-A1 was cultured in 75 mg/L of MSM for 5 days. Following this, the culture was centrifuged at 8,500 rpm, and the supernatant was filtered through a 0.22 µm filter (Jinteng, Tianjin, China) for use in the TC group. The PC group contained MSM without TYL, while the NC group contained MSM with a 75 mg/L concentration of TYL. Equal volumes (1% vol/vol) of *Staphylococcus aureus* and *E. coli* were added to each of the three groups and incubated at 37°C. Three replicates were set up for each treatment. Bacterial growth was assessed by measuring absorbance at 600 nm using a spectrophotometer (MU701, Shimadzu, Kyoto, Japan).

### Genome sequencing of TYL-A1

The strain TYL-A1 was inoculated in TSB medium and cultured at 30°C with shaking at 135 rpm until logarithmic growth was achieved. The bacterial culture was then centrifuged at 4,000 × *g* for 10 minutes at 4°C. The supernatant was discarded, and the pellet was resuspended in sterile water. This washing procedure was repeated three times. The bacterial pellet was collected into 1.5 mL EP tubes, sealed with sealing film, frozen in liquid nitrogen, and stored at −80°C. For 16S rDNA sequencing, a sample from the same bacterial batch was used. Genomic DNA was extracted by sodium dodecyl sulfate method (38). The harvested DNA was detected by agarose gel electrophoresis and quantified by Qubit 2.0 fluorometer. Whole-genome sequencing of the stained strain TYL-A1 was performed using the Nanopore PromethION platform and Illumina NovaSeq PE150 at Beijing Novozymes Bioinformatics Co. The whole-genome sequence of strain TYL-A1 was predicted using Unicycler software. The genes in the genome of strain TYL-A1 were predicted by GeneMarkS software and further annotated by Nr, SwissProt, COG, KEGG, and GO databases. A circular genome map of strain TYL-A1 was generated using Circos software (39) (accession number PRJNA949861).

### Application to bovine wastewater

The wastewater used was fresh and unfermented, obtained after solid-liquid separation. Initially, the wastewater was allowed to stand, and the supernatant was then filtered through a 0.45 µm membrane filter. The filtered wastewater was diluted 20-fold and autoclaved at 121°C for 30 minutes. In the test group, the bacterial suspension was added to the treated wastewater, while the control group received no bacterial strain and contained metal ions. Three replicates were set up for each treatment.

## RESULTS AND DISCUSSION

### Degradation characteristics of TYL by strain TYL-A1

**Fig 1:**
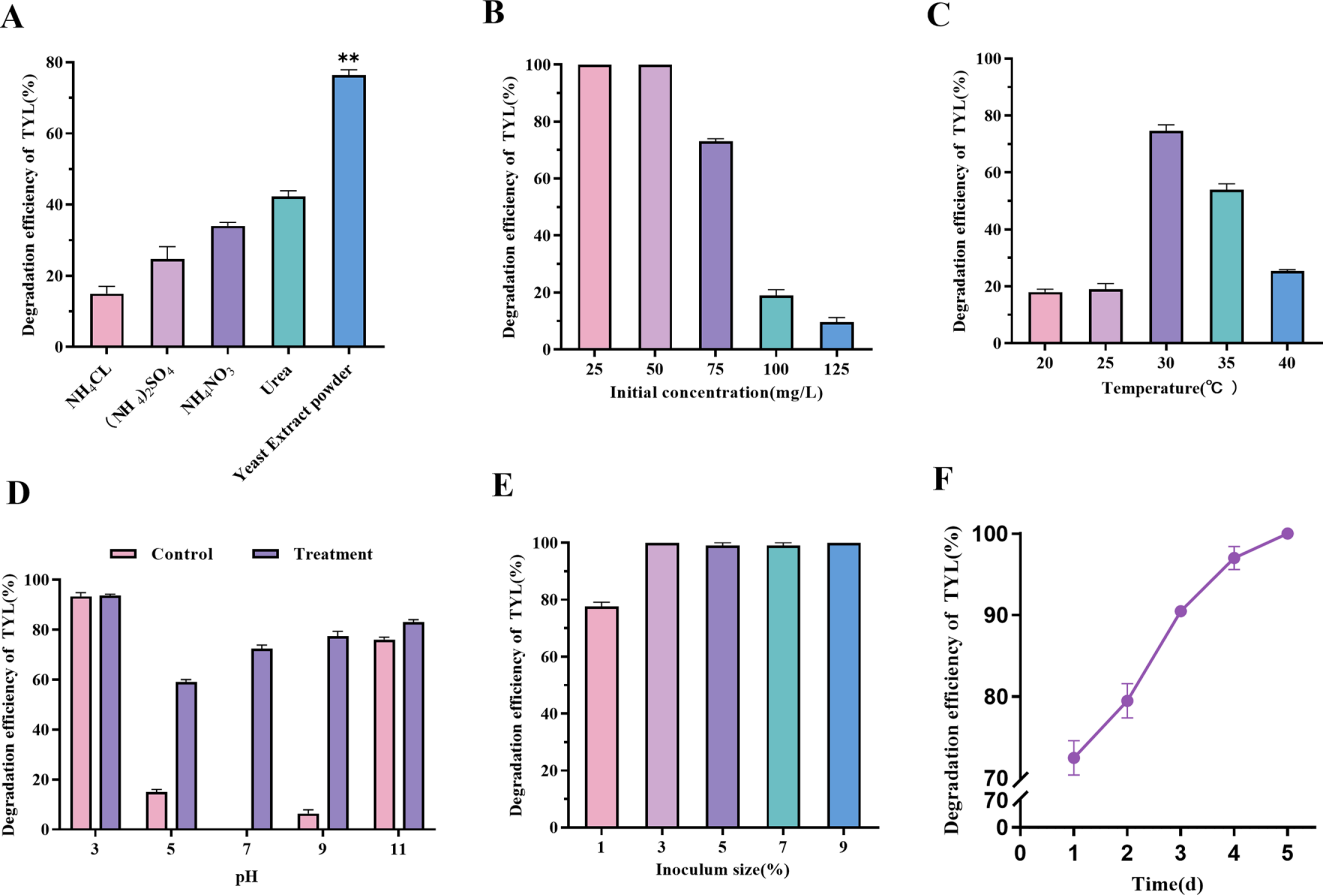
Effects of different factors on the degradation of TYL by strain TYL-A1: (**A**) effects of different nitrogen sources, (**B**) initial TYL concentrations, (**C**) temperatures, (**D**) pH values, (**E**) inoculation size, and (**F**) degradation under optimal conditions. Data points are means average: **P < 0.01.

The effects of the nitrogen source, initial concentration, temperature, pH, and inoculum amount on the degradation of TYL were investigated. All four nitrogen sources tested significantly promoted TYL degradation, with the highest degradation rate of 74.74% observed when using yeast leaching powder (Fig. 1A), so the nitrogen source in MSM was replaced with yeast leaching powder. The degradation rate of TYL by TYL-A1 decreased with increasing initial concentration. TYL-A1 completely degraded 50 mg/L of TYL and achieved a 72.78% degradation rate for 75 mg/L of TYL. However, only 19.57% and 10.31% degradation were observed at concentrations of 100 mg/L and 125 mg/L, respectively (Fig. 1B). These results indicate a toxic excitatory effect at higher TYL concentrations. This phenomenon, where a cell or organism exhibits a biphasic dose-response to an environmental stimulus, suggests that low concentrations of TYL can serve as a carbon source, supporting TYL-A1 growth (40). In contrast, excessive TYL concentrations inhibit TYL-A1 growth, thereby reducing the degradation rate. This is consistent with findings from gentamicin biodegradation studies by FZC3 (41).

Temperature and pH significantly influence the degradation of TYL. The degradation efficiency of TYL by strain TYL-A1 increased with rising temperature up to 30°C, after which it declined (Fig. 1C). Maximum degradation reached 74.09% at 30°C, while degradation at 35°C was 53.75%. These results indicate that temperatures between 30°C and 35°C accelerate the biodegradation of TYL. At temperatures below 30°C, biodegradation rates were slower, ranging from 18.12% to 17.43%. Both excessively high and low temperatures adversely affect the degradation efficiency of TYL-A1. Temperature plays a critical role in regulating microbial metabolism and enzyme activity. Optimal temperatures enhance microbial activity, whereas temperature fluctuations can reduce enzyme activity, thereby impacting degradation. The pH of the environment also affects microbial growth and metabolism, as microorganisms lack mechanisms to regulate their internal pH (42). In addition, pH impacts TYL stability; highly acidic or alkaline conditions promote natural degradation of TYL (43). Optimal degradation was observed at pH 3 and 11, with natural degradation rates of 91.33% and 76.95%, respectively. The degradation efficiency of TYL-A1 reached 70% at pH 7 (Fig. 1D). The bacterial cytoplasm, composed of water, salt, and other organic molecules, accumulates organic acids and polyamines, which affect the internal pH balance and subsequently influence bacterial growth, metabolic activities, and degradation efficiency (44). The strain maintains effective degradation capabilities within the pH range of 5–9.

The degradation of TYL with inoculation of 1%, 3%, 5%, 7%, and 9% of the culture was 77.37%, 99.71%, 99.73%, 99.69%, and 100%, respectively (Fig. 1E). The degradation rate increased with the inoculum concentration from 1% to 3%, likely due to the increased bacterial population shortening the logarithmic growth phase and accelerating TYL degradation (45). However, when the inoculum concentration exceeded 3%, there was no significant increase in the degradation rate. This may be attributed to competition among the microorganisms for TYL as the sole carbon source, leading to nutrient limitations for bacterial growth (46). An inoculation effect, characterized by a decrease in antibiotic efficiency with increasing bacterial population density, may also contribute to this observation (47). Due to cost considerations, a 3% inoculum was selected for further experimental studies.

In summary, the optimal degradation conditions (30°C, pH 7.0, 3% inoculum) achieved 99.68% degradation of TYL after 5 days (Fig. 1F). The strain TYL-A1 demonstrated effective TYL degradation capabilities. Notably, this strain, which was identified in this study, has not been previously reported for its ability to degrade TYL. Its degradation performance exceeds that of previously reported strains, including the acid-producing *Klebsiella* spp. isolated by Zhang et al. (48), *Pasteurella bacillus* reported by Hao et al. (49), *Citrobacter citriodora* screened by Ma et al. (50), and *Burkholderia vinelandii* discovered by Wang et al. (51).

A first-order kinetic equation was used to model the TYL degradation curve (Table S2). The regression coefficient (*R*²) for strain TYL-A1 was 0.9417. The calculated half-life of TYL in the presence of TYL-A1 was 0.8862 days or approximately 21 hours. In comparison, previous studies have reported the average half-life of TYL in feces to be between 4 and 8 days (52). This indicates that TYL-A1 significantly reduces the half-life of TYL, thereby accelerating its degradation.

### Degradation of TYL by strain TYL-A1 in the presence of metal ions

**Fig 2:**
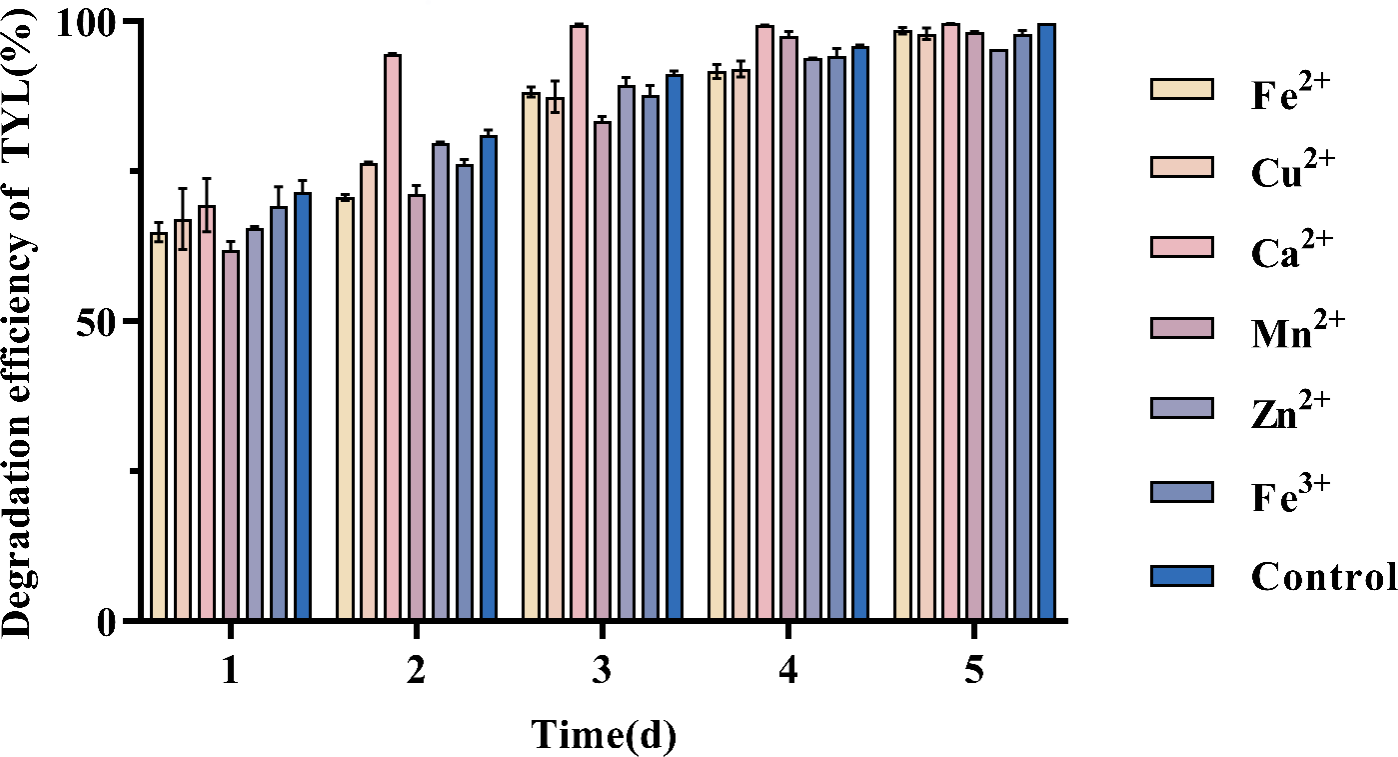
Degradation of TYL by strain TYL-A1 in the presence of metal ions.

Antibiotics ingested by livestock and poultry are often not fully absorbed and are excreted in feces, re-entering the environment and potentially impacting both animal health and ecological systems. Therefore, the degradation of antibiotics in feces is crucial. To assess the degradation capability of strain TYL-A1 in the presence of metal ions, TYL-A1 was added to MSM with various metal ions and TYL. A blank control group without added bacteria showed stable TYL levels. As shown in Fig. 2, after 5 days, metal ions such as Fe²^+^, Ca²^+^, Zn²^+^, Mn²^+^, Cu²^+^, and Fe³^+^ had no significant effect (*P* > 0.01) on the degradation of TYL by TYL-A1. Among these, the Ca²^+^ experimental group significantly accelerated degradation over 2–3 days (*P* < 0.01), achieving a 94.5% degradation of TYL within 2 days. The presence of Zn²^+^ inhibited degradation by strain TYL-A1, though this effect was not statistically significant (*P* > 0.01). The enhancement in degradation by Ca²^+^ may be attributed to co-metabolism, where the presence of a secondary substrate enhances the degradation of the primary compound (53). Calcium ions may support bacterial metabolism by maintaining osmotic pressure and promoting enzyme activity (54). Similarly, Fe³^+^ has been reported to accelerate the degradation of other contaminants, such as hygromycin, by Pseudomonas (55).

The results indicate that TYL-A1 is resistant to the effects of Fe²^+^, Ca²^+^, Zn²^+^, Mn²^+^, Cu²^+^, and Fe³^+^. Generally, metal ions can inhibit bacterial growth due to their toxic effects. However, TYL-A1 demonstrates an ability to adapt to metal and antibiotic co-pollution environments, achieving effective degradation. Future studies could explore whether TYL-A1 can degrade metal ions to better adapt to real environmental conditions. This study highlights the strain’s degradation capabilities in metal-antibiotic composite pollution scenarios, providing a theoretical basis for addressing metal-antibiotic co-pollution in the environment.

### Substrate utilization patterns of TYL-A1

**Fig 3:**
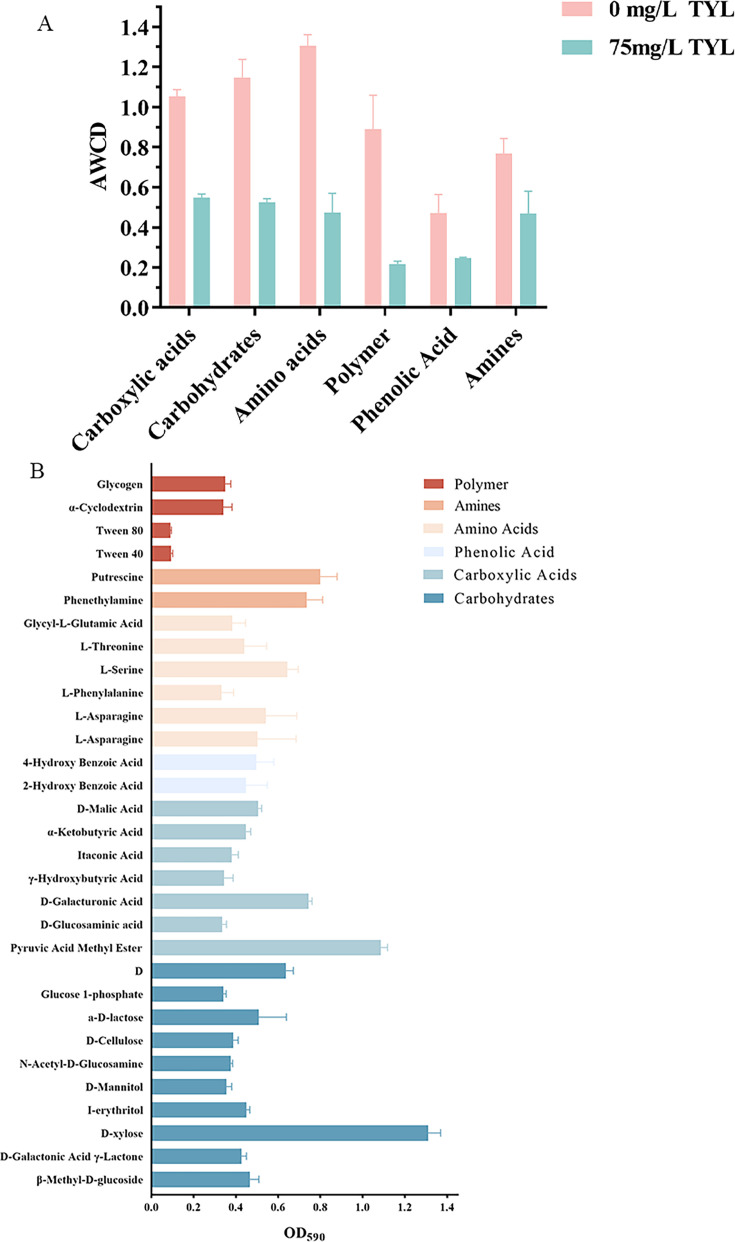
The carbon source utilization ability of strain TYL-A1 was analyzed by Biolog ecological plate when the concentration of TYL was 0 mg/L and 75 mg/L, respectively: (**A**) capacity to utilize six types of carbon sources and (**B**) OD_590_ values of 31 carbon substrates.

The AWCD was used as an indicator of metabolic activity. The addition of TYL affected AWCD, with lower AWCD values indicating reduced carbon source utilization and metabolic activity in TYL-treated TYL-A1. On day 10, the carbon source utilization pattern of TYL-A1 in the presence of TYL was examined. The utilization capacities of six categories of carbon sources are shown in Fig. 3A. The results indicated that carboxylic acids were the primary carbon sources utilized by TYL-A1, followed by carbohydrates, while polymers and phenolic acids were utilized to a lesser extent. The differences in microbial community metabolic functions were inferred from the OD values of color changes in reaction wells containing 31 different carbon sources (56). Figure 3B illustrates the OD_590_ values for all 31 carbon sources, with alanine methyl ester and D-xylose showing the highest OD_590_ values for carboxylic acids and carbohydrates, respectively. These compounds represent the preferred carbon sources for TYL-A1 and could be selected as co-substrates to enhance TYL biodegradation in future studies.

### Degradation of TYL by bacteria: intracellular and extracellular enzymes

**Fig 4:**
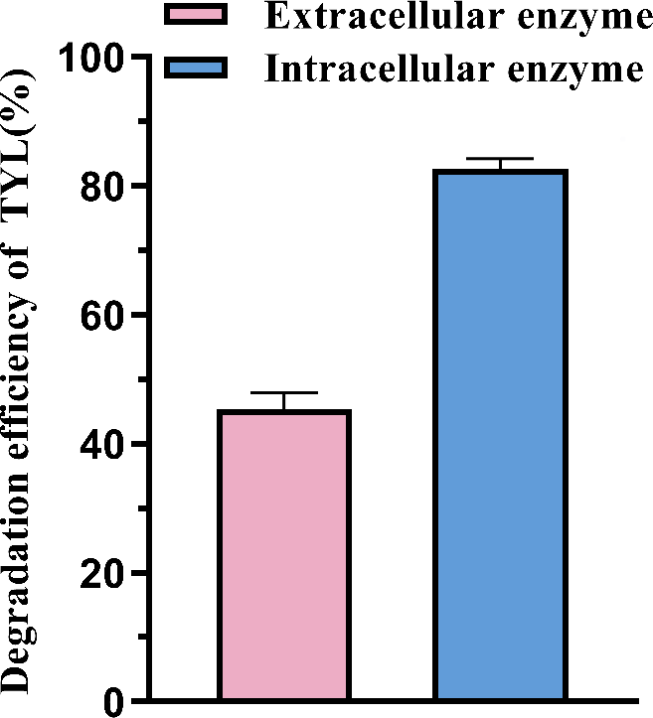
Effect of TYL degradation by intracellular and extracellular enzymes of strain TYL-A1.

Bacteria are capable of producing both intracellular and extracellular enzymes, which facilitate the degradation of organic pollutants through enzymatic reactions. As shown in Fig. 4, intracellular enzymes are more effective than extracellular enzymes in degrading TYL. Extracellular enzymes can directly interact with TYL in the environment, promoting its degradation through enzymatic reactions. For the degradation of TYL, the primary enzymes involved are likely macrolide-inactivating enzymes, hydrolases, and oxidoreductases, all of which are predominantly intracellular. In conclusion, under the same initial conditions, the biodegradation of TYL involves a combination of intracellular and extracellular enzymes, with intracellular enzymes demonstrating a higher efficiency in degrading TYL, achieving a degradation rate of 80.438%. However, the types of specific enzymes and their mechanism of action need further study. This finding aligns with previous studies; for example, Shao et al. (57) reported that both intracellular and extracellular enzymes produced by *Klebsiella* spp. were capable of degrading tetracycline, and Wang et al. (58) found that intracellular enzymes extracted and purified from *Burkholderia vietnamiensis* strains were effective in degrading TYL.

### TYL degradation products and pathways

**Fig 5:**
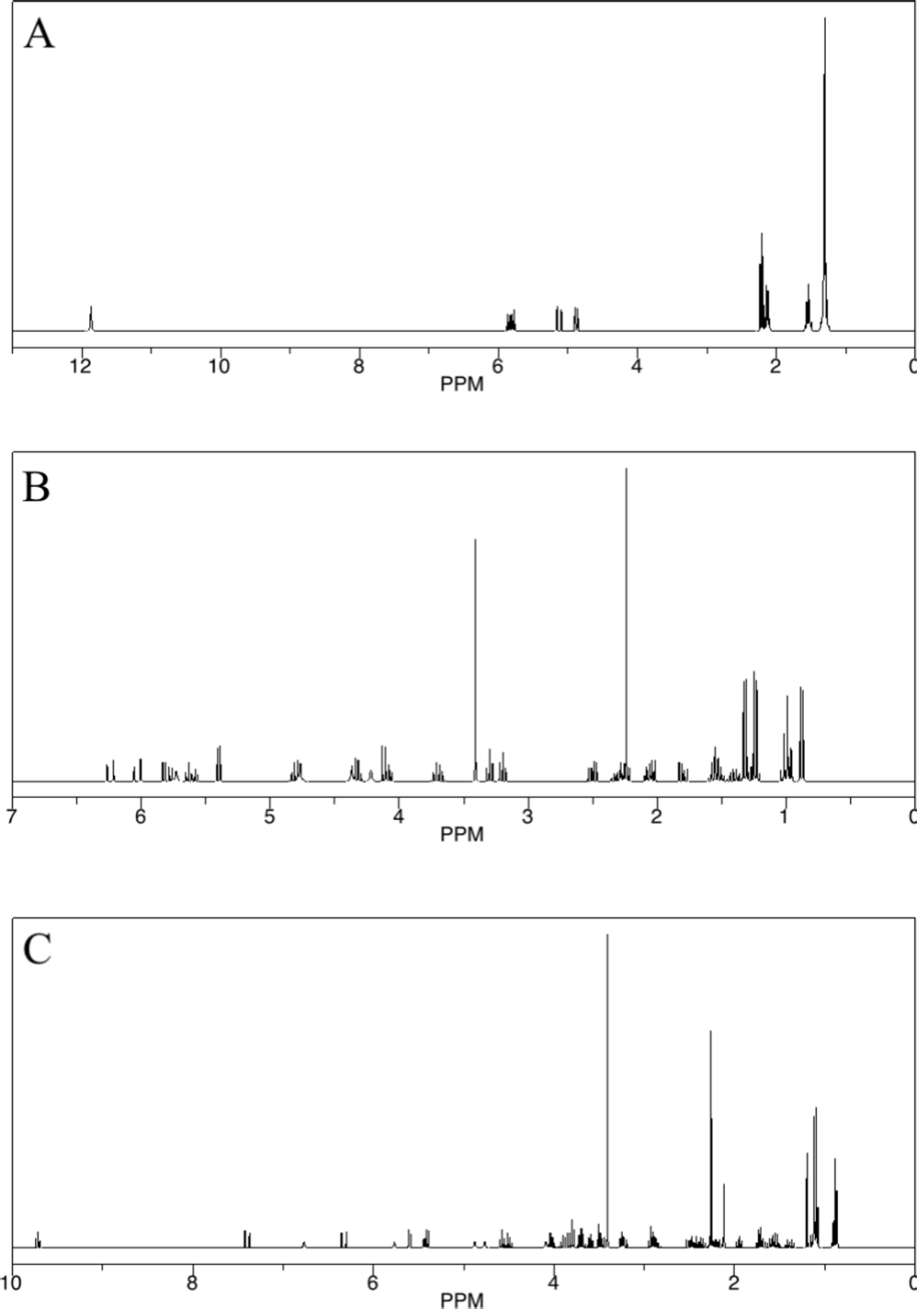
NMR spectra of degradation products: (A) TYL, (B) T1, and (C) T2.

**Fig 6:**
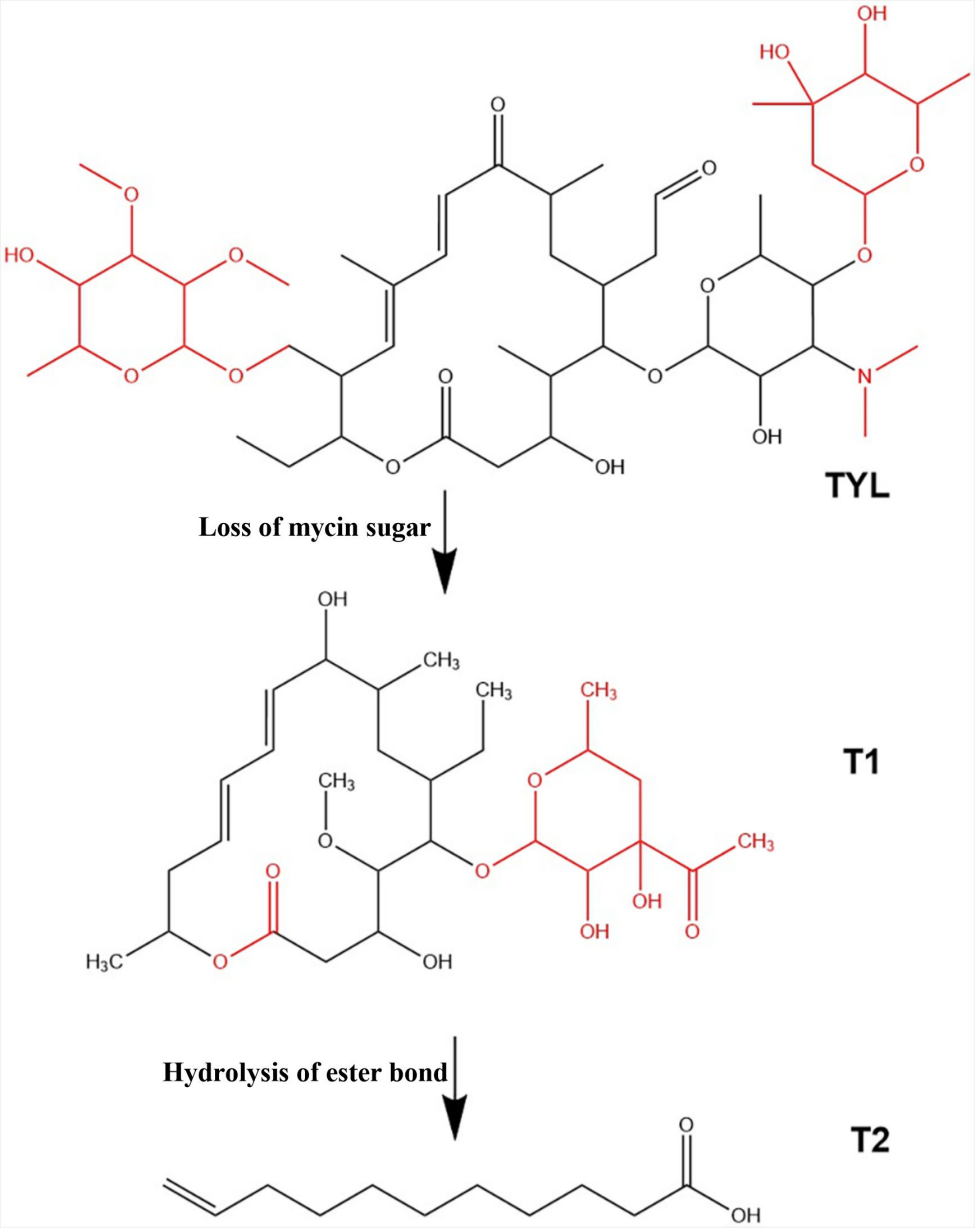
Metabolic pathway of TYL degradation by strain TYL- A1.

The intermediates of TYL degradation by strain TYL-A1 were analyzed and identified using liquid chromatography-mass spectrometry (LC-MS). The measured mass, compound structure, and elemental composition of the samples were compared with the HMDB database to obtain annotated information on the degradation products. The results of this analysis are presented in Table S3. Mass spectrometry revealed that the substance with a retention time of 6.102 minutes and an *M*/*Z* ratio of 898.52 was the substrate TYL. Two potential metabolites were detected: T1 with a retention time of 5.1359 minutes and T2 with a retention time of 2.9098 minutes. The mass-to-charge ratios for these metabolites were 556.32 and 223.11, respectively. The predicted NMR spectra for these metabolites are shown in Fig. 5. The biotransformation pathway was inferred using ChemDraw 16.0 and is illustrated in Fig. 6. Strain TYL-A1 initially acts on the penicillin sugar, removing some fragments to produce T1. T1 subsequently loses a sugar moiety, and its ester bond is hydrolyzed, leading to the breaking of the macrocycle. Further oxidation occurs, resulting in the cleavage of two double bonds and the formation of a new double bond, producing small-molecule substance T2. The initial removal of the amycin sugar is consistent with the findings of Yang et al. (59). The subsequent lactone hydrolysis is also supported by the literature, indicating that certain bacterial esterases can hydrolyze ester bonds in macrolide antibiotics (60). However, the product obtained in this study differs from previously reported metabolites (51, 61) and has a significantly smaller structure.

### Biological toxicity of degradation products

**Fig 7:**
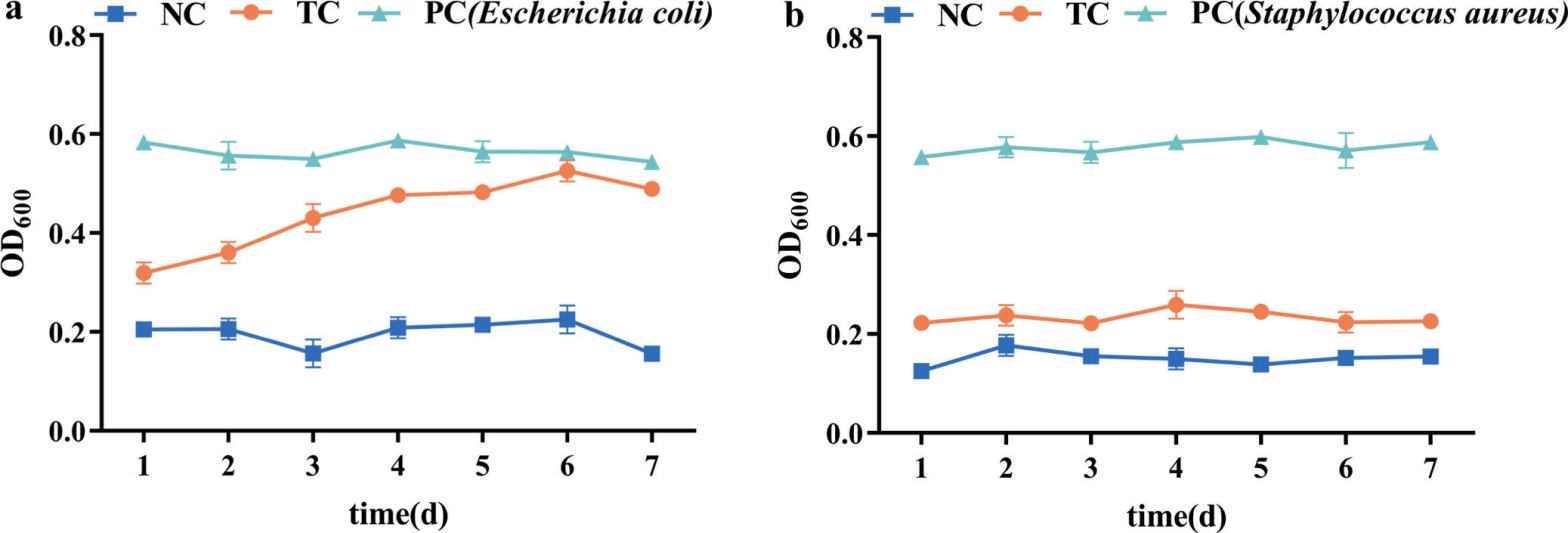
Bacterial inhibition by degradation products of strain TYL-A1: (**A**) *Escherichia coli* and (**B**) *Staphylococcus aureus.*

Although strain TYL-A1 efficiently degrades TYL, it is crucial to evaluate the biotoxicity of the degradation products to ensure that the degradation is both necessary and beneficial. As shown in Fig. 7, the growth of *Staphylococcus aureus* and *Escherichia coli* in the biodegradation-treated group (TYL-A1) was superior to that in the non-treated group (TYL) at various degradation times. This indicates that the degradation products generated by TYL-A1 are significantly less toxic than the parent compound. However, the growth rate of *S. aureus* was slower compared to *E. coli*, likely due to TYL’s greater inhibitory effect on gram-positive bacteria. Despite this, the biodegradation-treated group still showed superior growth compared to the non-treated group. These results demonstrate that TYL-A1 can successfully detoxify TYL, producing products with lower toxicity. This also suggests that microorganisms can effectively reduce the toxicity of TYL in the environment, offering significant advantages. The strains screened in this study indicate a novel pathway for TYL biotransformation, highlighting the effectiveness and significance of TYL-A1 in TYL biodegradation. Previous studies have shown similar results, such as the lower toxicity of CTC degradation products by LZ01 (62) and oxytetracycline (OTC) degradation products by OTC-16 (28). In summary, microbial degradation of antibiotics like TYL in the environment is more complete and offers greater advantages in terms of bioactivity compared to advanced oxidation, adsorption, and other abiotic degradation methods.

### Genome analysis of strain TYL-A1

**Fig 8:**
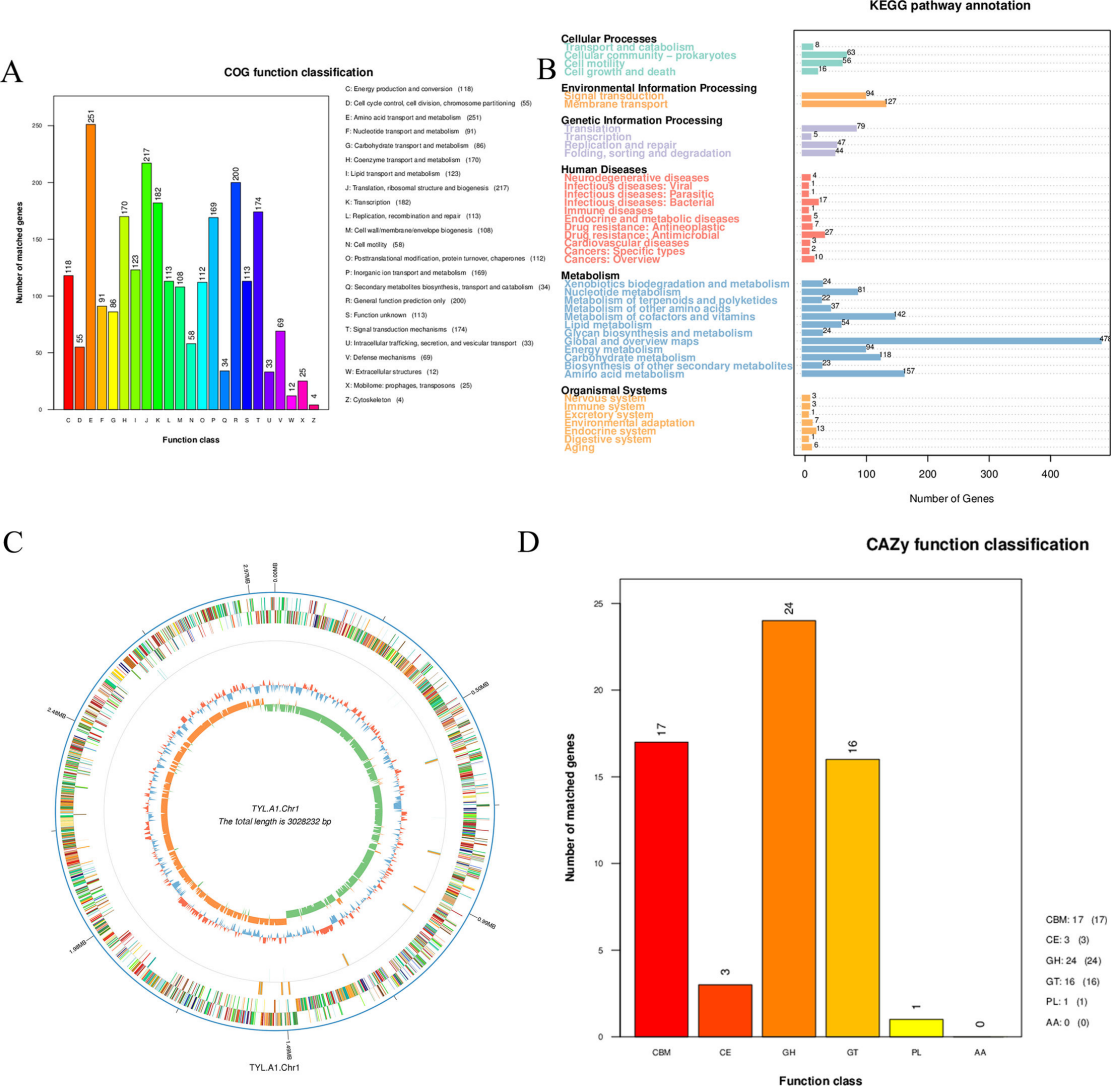
TYL-A1 gene function annotation. (**A**) COG pathway classification: the abscissa represents the COG functional type, and the ordinate represents the number of genes on the annotation. (**B**) KEGG metabolic pathway classification and the number on the bar chart represents the number of genes on the annotation, and the coordinate axis is the code of each functional class in the database. (**C**) Genome-wide map: the outermost circle is the position coordinates of the genome sequence. From the outside to the inside, it is the result of gene function annotation, ncRNA, and genome GC content. The blue part inward indicates that the GC content in this region is lower than the average GC content of the whole genome. The red part outward is the opposite. The genome GC skew value: window (chromosome length/1,000) bp, step length (chromosome length/1,000) bp, and the specific algorithm is G − C/G + C. The green part inward indicates that the content of G in this region is lower than the content of C, and the orange part outward is the opposite. (**D**) CAZy functional classification: the abscissa is the classification type of CAZy database, and the ordinate is the number of genes on the annotation.

Starting from the valid data after QC of each sample, genome assembly was performed using Unicycler (63) software using second-generation and third-generation data. The whole-genome sequencing of strain TYL-A1 has been submitted to the GenBank database. The chromosomal sequence of strain TYL-A1 was assembled into a circular genome, as shown in Fig. 8C. The total sequence length was 3,028,232 bp, with a GC content of 36.67% and 3,027 coding genes. One plasmid was identified (Fig. S2), with a length of 52,604 bp, a GC content of 35.17%, and 61 coding genes. The TYL-A1 chromosome contains 12 gene islands, 6 prophages, 2 CRISPRs, 187 repetitive sequences, and 109 non-coding RNAs. Only one prophage was present on the plasmid. To investigate the metabolism and degradation mechanisms of strain TYL-A1, the genome was analyzed using the COG, KEGG, GO, and CAZy databases. As shown in Fig. S1, biological processes and molecular functions play a major role in GO annotation, with metabolic processes and cellular processes actively involved in biological processes. Catalytic activity and binding are predominant in molecular functions, while cellular components and cellular fractions play key roles in cellular components. These results indicate that strain TYL-A1 is functionally rich, with strong metabolic functions and catalytic activity. The KEGG analysis (Fig. 8B) revealed that 1,905 genes on the chromosome and 2 genes (Fig. S2) on the plasmid were classified and annotated into the KEGG pathways. Previous studies suggest that genes related to terpene and polyketide metabolism, as well as sugar metabolism, may be involved in the biodegradation of TYL. The disruption of the TYL macrolide bond by proteins encoded by these genes (64) supports the LC-MS inferred degradation pathway observed in this study. Additionally, 24 genes were annotated in the heterologous biodegradation and metabolism pathway in the KEGG database, indicating that strain TYL-A1 can degrade not only TYL but also other organic pollutants, demonstrating significant research value and application potential. A total of 2,228 genes from the chromosome of strain TYL-A1 were annotated according to COG functional classification. As shown in Fig. 8A, 23 functions were classified, with the highest percentage being E (amino acid transport and metabolism) (251/2,228), followed by J (translation, ribosome, and biogenesis) (217/2,228), and R (general function prediction) (200/2,228). Eighteen genes were annotated to the plasmid (Fig. S2), with L (replication, recombination, and repair) having the highest percentage (6/18). As shown in Fig. 8D, strain TYL-A1 contained 17 carbohydrate-related genes , 24 glycoside hydrolases (GHs), 16 glycosyltransferases (GTs), 3 polysaccharide cleavage enzymes, and 1 polysaccharide lyase, with no oxidoreductases . Previous studies have shown that GHs and GTs degrade TYL by deglycosylation and play a key role in hydrolyzing the remaining macromolecular materials (65). Combined with LC-MS analysis, it suggests that relevant genes coding for GHs and GTs in strain TYL-A1 may be involved in the degradation of TYL by first acting on the mycosaccharides. Furthermore, the genome of strain TYL-A1 contains ARGs for various antibiotics (Table S3), with a total of 21 classes of drugs annotated, including aminoglycosides, quinolones, macrolides, glycopeptides, tetracyclines, and phenols. The strain exhibits four main resistance mechanisms: efflux of antibiotics, target displacement of antibiotics, target alteration of antibiotics, and inactivation of antibiotics (66). The resistance of strain TYL-A1 to TYL is attributed to the presence of macrolide resistance genes *mtrA*, *efrB*, *vmlR*, *MexD*, *ErmB*, and *mphB*. Previous studies have shown that *mphB* degrades macrolide antibiotics by encoding a 2′-phosphotransferase, which transfers r-phosphate on ATP to macrolide 2′-OH, thereby inhibiting antibiotic binding to the ribosome and inactivating it (67).

### Degradation of TYL in farm wastewater by TYL-A1

**Fig 9:**
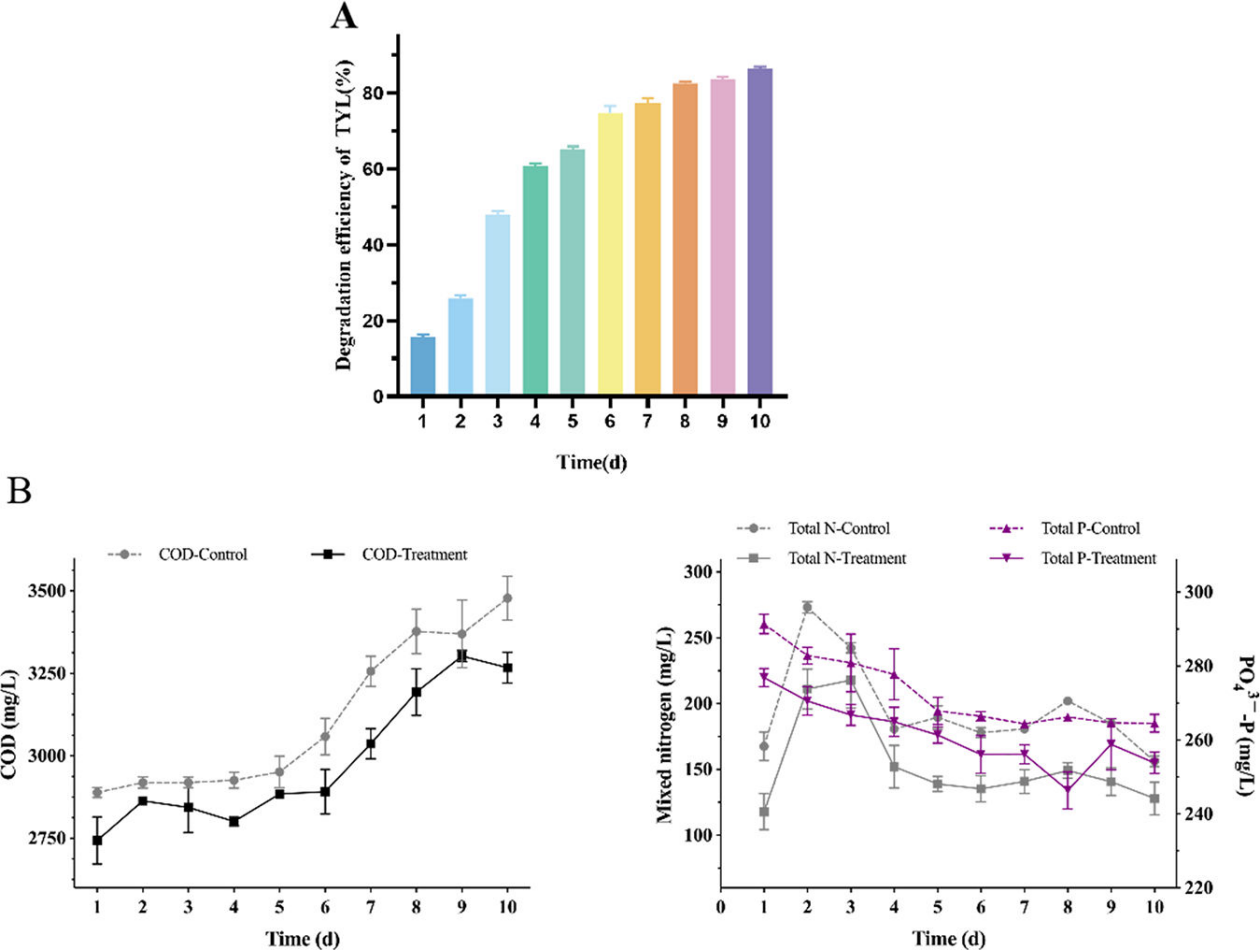
Degradation effect in wastewater: (**A**) changes in TYL and (**B**) changes in wastewater indicators during degradation: COD, TN, TP. TN, total nitrogen; TP, total phosphorus.

Breeding cattle wastewater contains high levels of chemical oxygen demand (COD), phosphorus (P), and nitrogen (N). Rapid and efficient remediation of such wastewater is crucial for environmental protection (59). To investigate the impact of TYL-A1 on TYL degradation in a simulated setting, TYL-A1 was introduced into diluted effluent from cow farms. After solid-liquid separation, TYL-A1 was added to the wastewater, and application experiments were conducted under laboratory conditions. Figure 9 shows the changes in TYL, COD, N, and P levels in the wastewater following the application of TYL-A1. The degradation rate of TYL in bovine wastewater increased rapidly, reaching 47% within the first 3 days. The rate then leveled off, reaching 80% after 8 days and finally 86.19% after 10 days (Fig. 9A). The degradation rate of TYL in wastewater was lower than in MSM, which can be attributed to two main reasons: (i) harsh and diverse environmental conditions: the complex and variable environment of wastewater can impact strain development and enzyme activity, slowing down the degradation process (68). (ii) Carbon catabolite metabolite inhibition: bacteria tend to prioritize carbon or energy sources that are readily biodegradable, inhibiting enzymes involved in utilizing other carbon and energy sources. The high organic content of wastewater introduces multiple substrates, which can provide additional energy and nutrients to microorganisms but may also cause inhibition of carbon catabolic metabolites (69). The results are consistent with those reported by Yang et al. (59).

COD is a crucial indicator of the overall amount of organic matter in water (70). The addition of antibiotics can increase COD in wastewater, and COD levels are also affected by bacterial growth and addition. Figure 9B illustrates that although COD increased over time, the total COD in the experimental group was lower than that in the control group after the addition of TYL-A1. This change in COD levels could be attributed to the growth of strain TYL-A1 and its degradation of TYL.

Total phosphorus (TP) in the experimental group was lower than in the control group and decreased over time. This reduction in TP may be attributed to the strains utilizing phosphorus as a nutrient, leading to a decrease in TP. Both groups exhibited a trend of TP increasing initially and then decreasing. Total nitrogen (TN) changes were influenced by nitrification and denitrification reactions (71). The results indicated that denitrification was more predominant than nitrification, resulting in a lower nitrogen concentration. TN gradually decreased in both groups, with the experimental group showing a lower TN compared to the control group. On the 10th day, the COD and TN levels in the wastewater treated with strain TYL-A1 were lower than those in the control group, and TP was also reduced. Overall, all indicators demonstrated that wastewater treated with strain TYL-A1 performed better than the control group, suggesting that the strain could have potential applications in aquaculture wastewater management.

### Conclusion

Microbial degradation of TYL is an effective bioremediation method. In this study, an efficient TYL-degrading bacterial strain TYL-A1 was isolated from TYL-contaminated soil. This strain demonstrated tolerance to TYL, temperature, pH, and metal ions. The addition of strain TYL-A1 significantly shortened the half-life of TYL. Both intracellular and extracellular enzymes contributed to the degradation process, and a new biometabolic pathway was proposed based on metabolite analysis by LC-MS. Whole-genome sequencing revealed that genes encoding GHs and GTs, along with genes involved in sugar metabolism, played roles in TYL degradation. This study elucidated the degradation mechanism of TYL and highlighted the potential of strain TYL-A1 to remove TYL from the environment, providing an important microbial germplasm resource for the bioremediation of TYL-contaminated environments.

## Data Availability

The raw data of genome sequencing in this project have been uploaded to NCBI under accession numbers PRJNA949861 and PRJNA949966.

## SUPPLEMENTAL MATERIAL

Supplemental material spectrum.00025-25-s0001.docx Tables S1 to S3; Fig. S1 and S2.

ASM does not own the copyrights to Supplemental Material that may be linked to, or accessed through, an article. The authors have granted ASM a non-exclusive, world-wide license to publish the Supplemental Material files. Please contact the corresponding author directly for reuse.

## References

[1] Liu J-L, Wong M-H. 2013. Pharmaceuticals and personal care products (PPCPs): a review on environmental contamination in China. Environ Int 59:208–224. doi:10.1016/j.envint.2013.06.012 23838081

[2] Tiseo K, Huber L, Gilbert M, Robinson TP, Van Boeckel TP. 2020. Global trends in antimicrobial use in food animals from 2017 to 2030. Antibiotics (Basel) 9:918. doi:10.3390/antibiotics9120918 33348801 PMC7766021

[3] Anadón A. 2006. WS14 The EU ban of antibiotics as feed additives (2006): alternatives and consumer safety. Vet Pharm Therapeutics 29:41–44. doi:10.1111/j.1365-2885.2006.00775_2.x

[4] Sarmah AK, Meyer MT, Boxall ABA. 2006. A global perspective on the use, sales, exposure pathways, occurrence, fate and effects of veterinary antibiotics (VAs) in the environment. Chemosphere 65:725–759. doi:10.1016/j.chemosphere.2006.03.026 16677683

[5] Zhang H, Luo Y, Wu L, Huang Y, Christie P. 2015. Residues and potential ecological risks of veterinary antibiotics in manures and composts associated with protected vegetable farming. Environ Sci Pollut Res 22:5908–5918. doi:10.1007/s11356-014-3731-9 25354434

[6] Huang Z, Mao C, Wei Y, Gu X, Cai Q, Shen X, Ding H. 2020. Analysis of the mutant selection window and killing of Mycoplasma hyopneumoniae for doxycycline, tylosin, danofloxacin, tiamulin, and valnemulin. PLoS One 15:e0220350. doi:10.1371/journal.pone.0220350 32544163 PMC7297357

[7] García-Sánchez L, Garzón-Zúñiga MA, Buelna G, Moeller-Chávez GE, Noyola A, Avilez-Flores M, Estrada-Arriaga EB. 2013. Occurrence of tylosin in swine wastewater in Mexico. Water Sci Technol 68:894–900. doi:10.2166/wst.2013.323 23985521

[8] Pokrant E, Trincado L, Yévenes K, Terraza G, Maddaleno A, Martín BS, Zavala S, Hidalgo H, Lapierre L, Cornejo J. 2021. Determination of five antimicrobial families in droppings of therapeutically treated broiler chicken by high-performance liquid chromatography-tandem mass spectrometry. Poult Sci 100:101313. doi:10.1016/j.psj.2021.101313 34298383 PMC8322472

[9] Li Y-X, Zhang X-L, Li W, Lu X-F, Liu B, Wang J. 2013. The residues and environmental risks of multiple veterinary antibiotics in animal faeces. Environ Monit Assess 185:2211–2220. doi:10.1007/s10661-012-2702-1 22692716

[10] Zhu D, Xiang Q, Yang X-R, Ke X, O’Connor P, Zhu Y-G. 2019. Trophic transfer of antibiotic resistance genes in a soil detritus food chain. Environ Sci Technol 53:7770–7781. doi:10.1021/acs.est.9b00214 31244079

[11] Shao S, Hu Y, Cheng J, Chen Y. 2018. Research progress on distribution, migration, transformation of antibiotics and antibiotic resistance genes (ARGs) in aquatic environment. Crit Rev Biotechnol 38:1195–1208. doi:10.1080/07388551.2018.1471038 29807455

[12] Fu Y, Zhu Y, Dong H, Li J, Zhang W, Shao Y, Shao Y. 2023. Effects of heavy metals and antibiotics on antibiotic resistance genes and microbial communities in soil. Process Saf Environ Prot 169:418–427. doi:10.1016/j.psep.2022.11.020

[13] Eniola JO, Kumar R, Barakat MA. 2019. Adsorptive removal of antibiotics from water over natural and modified adsorbents. Environ Sci Pollut Res Int 26:34775–34788. doi:10.1007/s11356-019-06641-6 31713137

[14] Nguyen LM, Nguyen NTT, Nguyen TTT, Nguyen TT, Nguyen DTC, Tran TV. 2022. Occurrence, toxicity and adsorptive removal of the chloramphenicol antibiotic in water: a review. Environ Chem Lett 20:1929–1963. doi:10.1007/s10311-022-01416-x 35369683 PMC8956153

[15] Cuerda-Correa EM, Alexandre-Franco MF, Fernández-González C. 2020. Advanced oxidation processes for the removal of antibiotics from water. an overview. Water (Basel) 12:102. doi:10.3390/w12010102

[16] Baaloudj O, Assadi I, Nasrallah N, El Jery A, Khezami L, Assadi AA. 2021. Simultaneous removal of antibiotics and inactivation of antibiotic-resistant bacteria by photocatalysis: a review. J Water Process Eng 42:102089. doi:10.1016/j.jwpe.2021.102089

[17] Gong Y, Wu Y, Xu Y, Li L, Li C, Liu X, Niu L. 2018. All-solid-state Z-scheme CdTe/TiO2 heterostructure photocatalysts with enhanced visible-light photocatalytic degradation of antibiotic waste water. Chem Eng J 350:257–267. doi:10.1016/j.cej.2018.05.186

[18] Pelalak R, Alizadeh R, Ghareshabani E, Heidari Z. 2020. Degradation of sulfonamide antibiotics using ozone-based advanced oxidation process: experimental, modeling, transformation mechanism and DFT study. Sci Total Environ 734:139446. doi:10.1016/j.scitotenv.2020.139446 32470661

[19] Lee H, Lee E, Lee C-H, Lee K. 2011. Degradation of chlorotetracycline and bacterial disinfection in livestock wastewater by ozone-based advanced oxidation. J Ind Eng Chem 17:468–473. doi:10.1016/j.jiec.2011.05.006

[20] Liu Y, Gao J, Zhao M, Fu X, Zhang Y, Zhang H. 2023. Removal of antibiotic resistant bacteria, genes and inhibition of plasmid-mediated horizontal transfer by peroxymonosulfate: efficiency and mechanisms. Chem Eng J 453:139728. doi:10.1016/j.cej.2022.139728

[21] Forouzesh M, Ebadi A, Aghaeinejad-Meybodi A. 2019. Degradation of metronidazole antibiotic in aqueous medium using activated carbon as a persulfate activator. Sep Purif Technol 210:145–151. doi:10.1016/j.seppur.2018.07.066

[22] Zakaria BS, Dhar BR. 2022. A review of stand-alone and hybrid microbial electrochemical systems for antibiotics removal from wastewater. Processes (Basel) 10:714. doi:10.3390/pr10040714

[23] Zheng W, Tsang C-S, So LY, Liu M, Leung Y-C, Lee LYS. 2019. Highly efficient stepwise electrochemical degradation of antibiotics in water by in situ formed Cu(OH)2 nanowires. Appl Catalysis B Environ 256:117824. doi:10.1016/j.apcatb.2019.117824

[24] Martínez-Costa JI, Rivera-Utrilla J, Leyva-Ramos R, Sánchez-Polo M, Velo-Gala I, Mota AJ. 2018. Individual and simultaneous degradation of the antibiotics sulfamethoxazole and trimethoprim in aqueous solutions by Fenton, Fenton-like and photo-Fenton processes using solar and UV radiations. J Photochem Photobiol A Chem 360:95–108. doi:10.1016/j.jphotochem.2018.04.014

[25] Furia F, Minella M, Gosetti F, Turci F, Sabatino R, Di Cesare A, Corno G, Vione D. 2021. Elimination from wastewater of antibiotics reserved for hospital settings, with a Fenton process based on zero-valent iron. Chemosphere 283:131170. doi:10.1016/j.chemosphere.2021.131170 34467949

[26] Guo X, Liu M, Zhong H, Li P, Zhang C, Wei D, Zhao T. 2020. Potential of Myriophyllum aquaticum for phytoremediation of water contaminated with tetracycline antibiotics and copper. J Environ Manage 270:110867. doi:10.1016/j.jenvman.2020.110867 32507744

[27] Yan Y, Chen Y, Xu X, Zhang L, Wang G. 2019. Effects and removal of the antibiotic sulfadiazine by eichhornia crassipes: potential use for phytoremediation. Bull Environ Contam Toxicol 103:342–347. doi:10.1007/s00128-019-02656-4 31222425

[28] Shi Y, Lin H, Ma J, Zhu R, Sun W, Lin X, Zhang J, Zheng H, Zhang X. 2021. Degradation of tetracycline antibiotics by Arthrobacter nicotianae OTC-16. J Hazard Mater 403:123996. doi:10.1016/j.jhazmat.2020.123996 33265032

[29] Ahmed MB, Zhou JL, Ngo HH, Guo W. 2015. Adsorptive removal of antibiotics from water and wastewater: progress and challenges. Sci Total Environ 532:112–126. doi:10.1016/j.scitotenv.2015.05.130 26057999

[30] Poynton HC, Robinson WE. 2018. Chapter 3.7 - Contaminants of emerging concern, with an emphasis on nanomaterials and pharmaceuticals, p 291–315. In Török B, Dransfield T (ed), Green chemistry. Elsevier.

[31] Müller MM, Nedielkov R, Arndt KM. 2022. Strategies for enzymatic inactivation of the veterinary antibiotic florfenicol. Antibiotics (Basel) 11:443. doi:10.3390/antibiotics11040443 35453195 PMC9029715

[32] Dong Z, Yan X, Wang J, Zhu L, Wang J, Li C, Zhang W, Wen S, Kim YM. 2022. Mechanism for biodegradation of sulfamethazine by Bacillus cereus H38. Sci Total Environ 809:152237. doi:10.1016/j.scitotenv.2021.152237 34890664

[33] Wang J, Zhang M, Cheng J, Han Y, Ma H, Jiang X, Liu Y. 2024. Molecular mechanism underlying the degradation of tetracycline by Apiotrichum loubieri MFZ-16. Environ Technol Innov 33:103489. doi:10.1016/j.eti.2023.103489

[34] Tan Z, Chen J, Liu Y, Chen L, Xu Y, Zou Y, Li Y, Gong B. 2021. The survival and removal mechanism of Sphingobacterium changzhouense TC931 under tetracycline stress and its’ ecological safety after application. Bioresour Technol 333:125067. doi:10.1016/j.biortech.2021.125067 33878498

[35] Zhao B, Wang Y, Zhang J, Wang L, Basang W, Zhu Y, Gao Y. 2024. Development and assessment of an immobilized bacterial alliance that efficiently degrades tylosin in wastewater. PLoS One 19:e0304113. doi:10.1371/journal.pone.0304113 38820335 PMC11142594

[36] Feng N-X, Yu J, Xiang L, Yu L-Y, Zhao H-M, Mo C-H, Li Y-W, Cai Q-Y, Wong M-H, Li QX. 2019. Co-metabolic degradation of the antibiotic ciprofloxacin by the enriched bacterial consortium XG and its bacterial community composition. Sci Total Environ 665:41–51. doi:10.1016/j.scitotenv.2019.01.322 30772572

[37] Li H, Dong T, Tao M, Zhao H, Lan T, Yan S, Gong X, Hou Q, Ma X, Song Y. 2024. Fucoidan enhances the anti-tumor effect of anti-PD-1 immunotherapy by regulating gut microbiota. Food Funct 15:3463–3478. doi:10.1039/D3FO04807A 38456333

[38] Lee YK, Kim HW, Liu CL, Lee HK. 2003. A simple method for DNA extraction from marine bacteria that produce extracellular materials. J Microbiol Methods 52:245–250. doi:10.1016/S0167-7012(02)00180-X 12459245

[39] Krzywinski M, Schein J, Birol I, Connors J, Gascoyne R, Horsman D, Jones SJ, Marra MA. 2009. Circos: an information aesthetic for comparative genomics. Genome Res 19:1639–1645. doi:10.1101/gr.092759.109 19541911 PMC2752132

[40] Surma R, Wojcieszyńska D, Karcz J, Guzik U. 2021. Effect of Pseudomonas moorei KB4 cells’ immobilisation on their degradation potential and tolerance towards paracetamol. Molecules 26:820. doi:10.3390/molecules26040820 33557429 PMC7915102

[41] Liu Y, Chang H, Li Z, Zhang C, Feng Y, Cheng D. 2016. Gentamicin removal in submerged fermentation using the novel fungal strain Aspergillus terreus FZC3. Sci Rep 6:35856. doi:10.1038/srep35856 27775038 PMC5075888

[42] Bhattacharya S, Das A, G M, K V, J S. 2011. Mycoremediation of congo red dye by filamentous fungi. Braz J Microbiol 42:1526–1536. doi:10.1590/S1517-838220110004000040 24031787 PMC3768715

[43] Feng C -q., Cheng D -m., Feng Y, Qi W -n., Jia Z -h., Weaver L, Liu Y -w., Li Z -j. 2020. Screening and degradation characteristics of a tylosin-degrading strain. J Integr Agric 19:1127–1136. doi:10.1016/S2095-3119(19)62764-4

[44] Chandrashekar V, Chandrashekar C, Shivakumar R, Bhattacharya S, Das A, Gouda B, Rajan SS. 2014. Assessment of acrylamide degradation potential of Pseudomonas aeruginosa BAC-6 isolated from industrial effluent. Appl Biochem Biotechnol 173:1135–1144. doi:10.1007/s12010-014-0923-1 24771288

[45] Yu C, Chen F, Zhao X, Guo T, Lin R, Qu M, Liu Q. 2011. Isolation, identification and degrading characteristics of phenol-degrading bacteria B3. Proceedings of the 2011 International Conference on Electrical and Control Engineering, p 3509–3512

[46] Dianatdar F, Etemadifar Z, Momenbeik F. 2024. Removal of amoxicillin and co-amoxiclav by newly isolated Stenotrophomonas maltophilia DF1. Int J Environ Sci Technol 21:9377–9390. doi:10.1007/s13762-024-05709-2

[47] Diaz-Tang G, Meneses EM, Patel K, Mirkin S, García-Diéguez L, Pajon C, Barraza I, Patel V, Ghali H, Tracey AP, Blanar CA, Lopatkin AJ, Smith RP. 2022. Growth productivity as a determinant of the inoculum effect for bactericidal antibiotics. Sci Adv 8:eadd0924. doi:10.1126/sciadv.add0924 36516248 PMC9750144

[48] Zhang T, Xu S, Lin H, Yang J, Zhao Z, Barceló D, Zheng H. 2022. Efficient degradation of tylosin by Klebsiella oxytoca TYL-T1. Sci Total Environ 847:157305. doi:10.1016/j.scitotenv.2022.157305 35839875

[49] Hao P, Lv Z, Wu S, Zhang X, Gou C, Wang L, Zhu Y, Basang W, Gao Y. 2023. Transcriptome profiling of Microbacterium resistens MZT7 reveals mechanisms of 17β-estradiol response and biotransformation. Environ Res 217:114963. doi:10.1016/j.envres.2022.114963 36471558

[50] Ma Y, Wang L, Liu L, Zhang X. 2015. Biodegradation of tylosin residue in pharmaceutical solid waste by a novel Citrobacter amalonaticus strain. Env Prog and Sustain Energy 34:99–104. doi:10.1002/ep.11961

[51] Wang Y, Ma Y, Ma L, Chang X, Sun R, Wang M, Zhang J. 2015. Study on microbial degradation pathway and products of tylosin residue in pharmaceutical waste. Huanjing Kexue Xuebao/Acta Scientiae Circumstantiae 35:491–498. doi:10.13671/j.hjkxxb.2014.1086

[52] Ramaswamy J, Prasher SO, Patel RM, Hussain SA, Barrington SF. 2010. The effect of composting on the degradation of a veterinary pharmaceutical. Bioresour Technol 101:2294–2299. doi:10.1016/j.biortech.2009.10.089 19944598

[53] Hao P, Wu S, Zhang X, Gou C, Wang Y, Wang L, Zhu Y, Basang W, Gao Y. 2022. Characterization and degradation pathways of Microbacterium resistens MZT7, a novel 17β-estradiol-degrading bacterium. Int J Environ Res Public Health 19:11097. doi:10.3390/ijerph191711097 36078812 PMC9518027

[54] Zheng T, Qian C. 2020. Influencing factors and formation mechanism of CaCO3 precipitation induced by microbial carbonic anhydrase. Process Biochem 91:271–281. doi:10.1016/j.procbio.2019.12.018

[55] Qi W, Long J, Feng C, Feng Y, Cheng D, Liu Y, Xue J, Li Z. 2019. Fe3+ enhanced degradation of oxytetracycline in water by pseudomonas. Water Res 160:361–370. doi:10.1016/j.watres.2019.05.058 31158618

[56] Li S, Lü T, Zhang X, Gu GH, Niu Y. 2013. Effect of Trichoderma longbrachiatum T2 on functional diversity of cucumber rhizomicrobes. J Environ Biol 34:293–299. 24620596

[57] Shao S, Hu Y, Cheng J, Chen Y. 2019. Biodegradation mechanism of tetracycline (TEC) by strain Klebsiella sp. SQY5 as revealed through products analysis and genomics. Ecotoxicol Environ Saf 185:109676. doi:10.1016/j.ecoenv.2019.109676 31539769

[58] Wang L-Q, Ma Y-L, Zhang J, Wang Y, Sun R-Z. 2017. Enzymatic degradation and metabolic product of tylosin residue using enzyme extracted from Burkholderia vietnamiensis strain. Env Prog and Sustain Energy 36:879–886. doi:10.1002/ep.12528

[59] Yang J, Zhao Z -q., Wang M, Yu K -f., Zhang T, Lin H, Zheng H -b. 2022. Biodegradation of tylosin in swine wastewater by Providencia stuartii TYL-Y13: performance, pathway, genetic background, and risk assessment. J Hazard Mater 440:129716. doi:10.1016/j.jhazmat.2022.129716 35952431

[60] Mitchell SM, Ullman JL, Teel AL, Watts RJ. 2015. Hydrolysis of amphenicol and macrolide antibiotics: chloramphenicol, florfenicol, spiramycin, and tylosin. Chemosphere 134:504–511. doi:10.1016/j.chemosphere.2014.08.050 25618189

[61] Zhang B, Wang M, Qu J, Zhang Y, Liu H. 2021. Characterization and mechanism analysis of tylosin biodegradation and simultaneous ammonia nitrogen removal with strain Klebsiella pneumoniae TN-1. Bioresour Technol 336:125342. doi:10.1016/j.biortech.2021.125342 34082338

[62] Zhang S, Wang J. 2022. Biodegradation of chlortetracycline by Bacillus cereus LZ01: performance, degradative pathway and possible genes involved. J Hazard Mater 434:128941. doi:10.1016/j.jhazmat.2022.128941 35462123

[63] Wick RR, Judd LM, Gorrie CL, Holt KE. 2017. Unicycler: resolving bacterial genome assemblies from short and long sequencing reads. PLOS Comput Biol 13:e1005595. doi:10.1371/journal.pcbi.1005595 28594827 PMC5481147

[64] Golkar T, Zieliński M, Berghuis AM. 2018. Look and outlook on enzyme-mediated macrolide resistance. Front Microbiol 9:1942. doi:10.3389/fmicb.2018.01942 30177927 PMC6109786

[65] Giedraitienė A, Vitkauskienė A, Naginienė R, Pavilonis A. 2011. Antibiotic resistance mechanisms of clinically important bacteria. Medicina (Kaunas) 47:137–146. 21822035

[66] Liu Y, Cai Y, Li G, Wang W, Wong PK, An T. 2022. Response mechanisms of different antibiotic-resistant bacteria with different resistance action targets to the stress from photocatalytic oxidation. Water Res 218:118407. doi:10.1016/j.watres.2022.118407 35453030

[67] Noguchi N, Tamura Y, Katayama J, Narui K. 1998. Expression of the mphB gene for macrolide 2′-phosphotransferase II from Escherichia coli in Staphylococcus aureus. FEMS Microbiol Lett 159:337–342. doi:10.1016/S0378-1097(98)00003-2 9503630

[68] Jiang Y, Brassington KJ, Prpich G, Paton GI, Semple KT, Pollard SJT, Coulon F. 2016. Insights into the biodegradation of weathered hydrocarbons in contaminated soils by bioaugmentation and nutrient stimulation. Chemosphere 161:300–307. doi:10.1016/j.chemosphere.2016.07.032 27441989 PMC4991617

[69] Hong X, Zhao Y, Zhuang R, Liu J, Guo G, Chen J, Yao Y. 2020. Bioremediation of tetracycline antibiotics-contaminated soil by bioaugmentation. RSC Adv 10:33086–33102. doi:10.1039/D0RA04705H 35694106 PMC9122622

[70] Aguilar-Torrejón JA, Balderas-Hernández P, Roa-Morales G, Barrera-Díaz CE, Rodríguez-Torres I, Torres-Blancas T. 2023. Relationship, importance, and development of analytical techniques: COD, BOD, and, TOC in water—An overview through time. SN Appl Sci 5:118. doi:10.1007/s42452-023-05318-7

[71] Liu Y, Zhu T, Ren S, Zhao T, Chai H, Xu Y, Peng L, Liu Y. 2022. Contribution of nitrification and denitrification to nitrous oxide turnovers in membrane-aerated biofilm reactors (MABR): a model-based evaluation. Sci Total Environ 806:151321. doi:10.1016/j.scitotenv.2021.151321 34743877

