# Supplementary material for: Efficient degradation of tylosin by Kurthia gibsonii TYL-A1: performance, pathway, and genomics study
Source: Microbiol Spectr. 2025 Apr 29;13(6):e00025-25. doi: 10.1128/spectrum.00025-25 (PMC12131772; doi:10.1128/spectrum.00025-25)
Supplement: Supplemental material — Tables S1 to S3; Fig. S1 and S2. [file spectrum.00025-25-s0001.docx]

**Efficient degradation of tylosin by K.gibsonii TYL-A1 : performance, pathway and genomics study**

Ye Wang^a^, Boyu Zhao^a^, Jingyi Zhang^a^ , Lingcong Kong^a^ , Inam Muhammad^b^,Xiaojun Liang^c^, Xiuzhen Yu ^d*^,Yunhang Gao^a*^

^a^College of Veterinary Medicine, Jilin Agricultural University, Changchun, 130118, China

^b^Department of Zoology, Shaheed Benazir Bhutto University Sheringal, Dir Upper 18050, Pakistan.

^c^Institute of Animal Science, Ningxia Academy of Agriculture and Forestry Sciences, Yinchuan, Ningxia 750002, China.

^d^Agricultural Mechanization Research Institute, Xinjiang Academy of Agricultural Sciences

* Corresponding author : Yunhang Gao, Xiuzhen Yu

E-mail address: gaoyunhang@163.com (Y. Gao) ; [yxzshz@126.com](mailto:yxzshz@126.com)

Tab.S1 Mobile phase elution gradient - positive ion mode

| Time (min) | Flow rate (mL/min) | A (%) | B (%) |
| --- | --- | --- | --- |
| 0 | 0.4 | 100 | 0 |
| 3 | 0.4 | 80 | 20 |
| 4.5 | 0.4 | 65 | 35 |
| 5 | 0.4 | 0 | 100 |
| 6.3 | 0.4 | 0 | 100 |
| 6.4 | 0.4 | 100 | 0 |
| 8 | 0.4 | 100 | 0 |

Tab.S2 Kinetic equations and kinetic parameters for degradation of TYL

| Fitting equation | k/h^-1^ | t_1/2_/d | R^2^ |
| --- | --- | --- | --- |
| Ln （Ct/C0） =-1.0301t + 0.2197 | -1.0301 | 0.8862 | 0.9417 |

Tab.S3 CARD partial annotation results

| 药物类别 | 数量 |
| --- | --- |
| Aminoglycoside antibiotic | 14 |
| Fluoroquinolone antibiotic | 10 |
| Macrolide antibiotic | 8 |
| Phenicol antibiotic | 8 |
| Lincosamide antibiotic | 8 |
| Glycopeptide antibiotic | 7 |
| Tetracycline antibiotic | 7 |
| Acridine dye | 6 |
| Disinfecting agents and intercalating dyes | 6 |
| Diaminopyrimidine antibiotic | 4 |


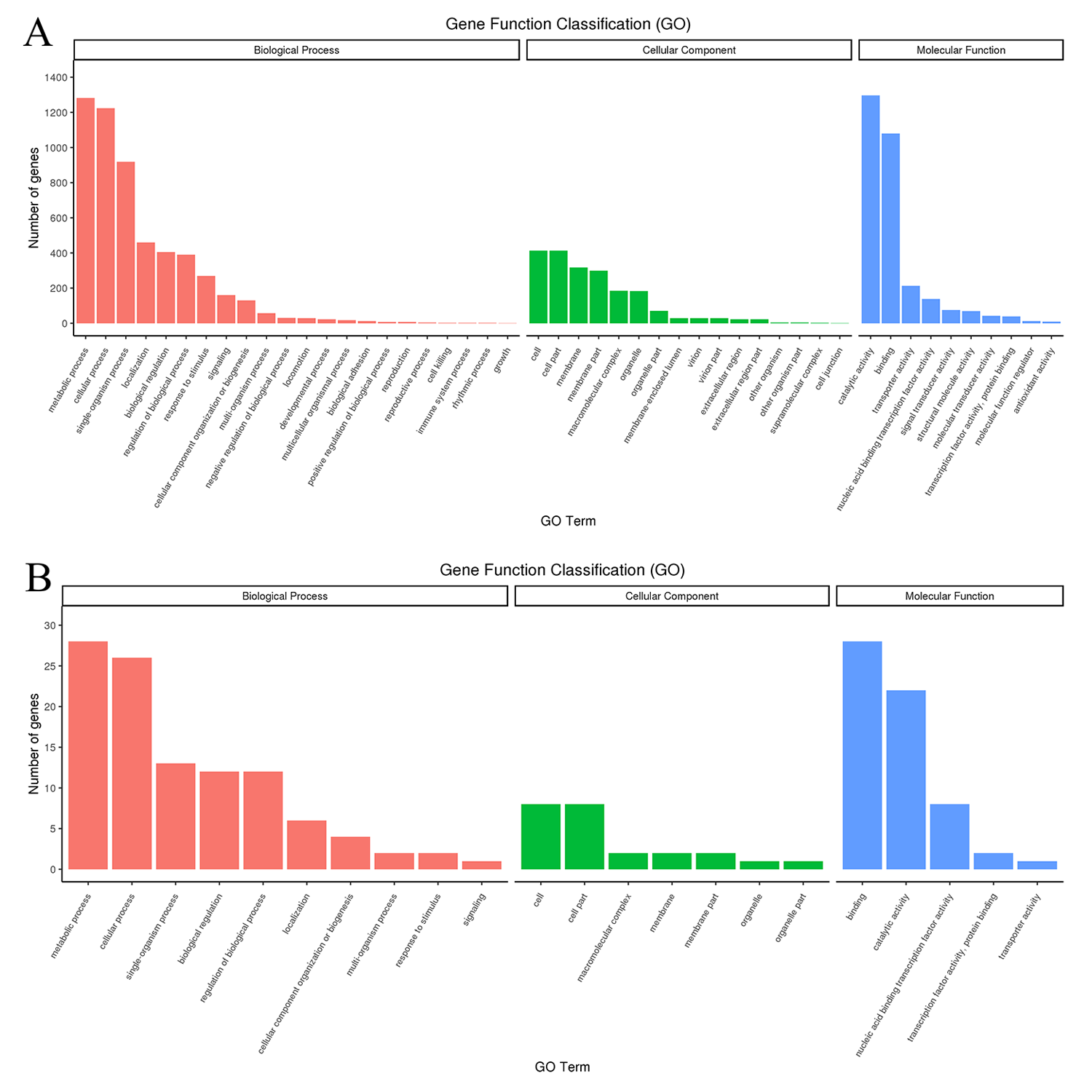


**Fig****. S1** GO function annotation statistics graph, A: Chromosome; B: Plasmid. Horizontal coordinates are the GO term at the next level of the three broad GO categories, and vertical coordinates are the number of genes annotated to that term . 3 different classifications represent the three basic classifications of GO terms (biological process, cellular component, and molecular function, from left to right).


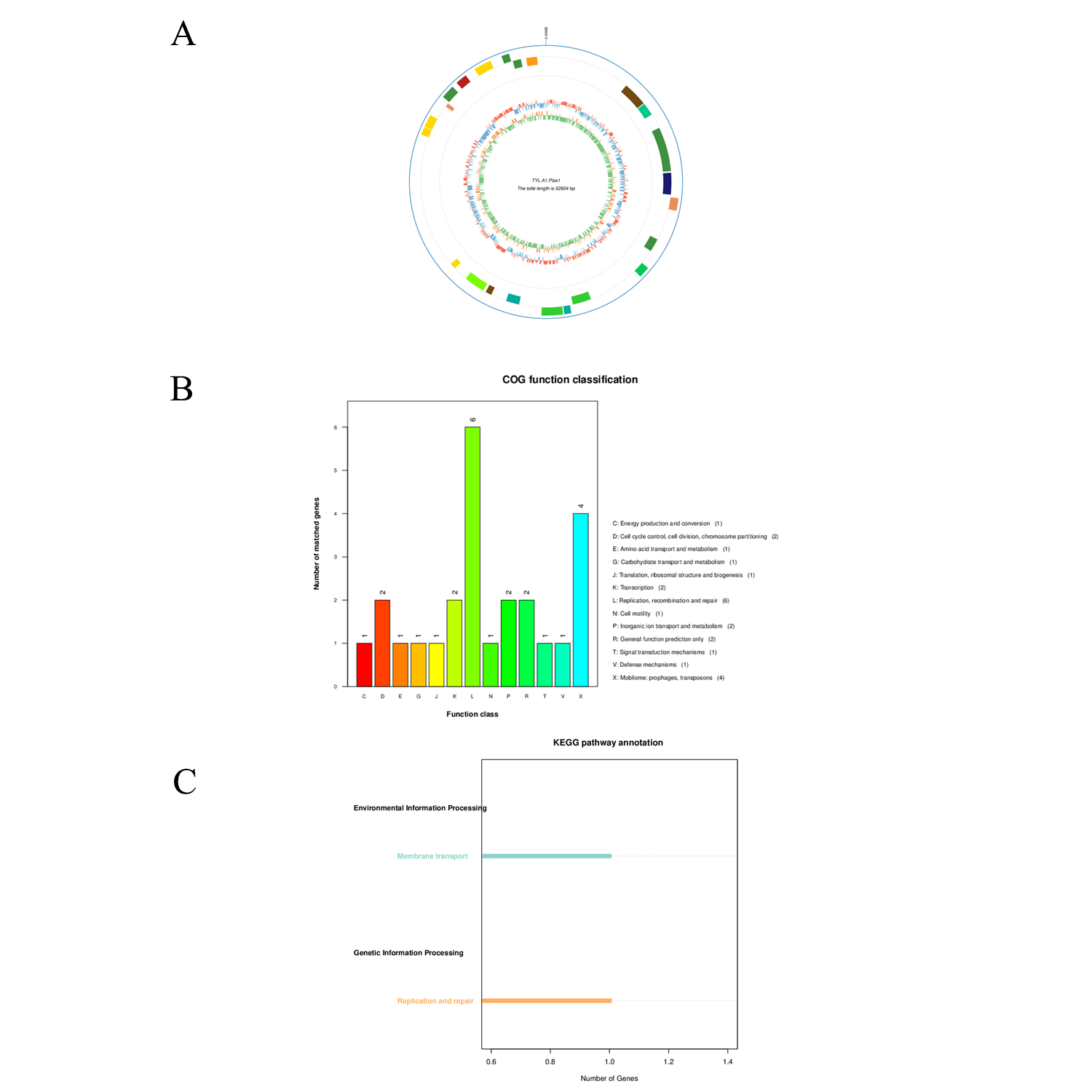


**Fig. S2** Functional annotation of TYL-A1 gene Plasmid (A) Genome-wide map ,the picture is from the outside to the inside, which is the COG functional annotation classification gene ( the arrow clockwise indicates the positive chain coding ), the genome sequence position coordinate, and the genome GC content : the window is 500 bp, and the step size is 20 bp. The blue part indicates that the GC content in the region is lower than the average GC content of the whole genome, and the red part is opposite, and the higher the peak value indicates the greater the difference from the average GC content, and the genome GC skew value : the window is 500 bp, and the step size is 20 bp. The specific algorithm is G-C / G + C. The green part indicates that the content of G in the region is lower than that of C, and the orange part is opposite. (B) COG pathway classification, the abscissa represents the COG functional type, and the ordinate represents the number of genes on the annotation. (C) KEGG metabolic pathway classification, the number on the bar graph represents the number of genes on the annotation ; the coordinate axis is the code of each functional class in the database.
